# Country-Wide Analysis of Systemic Factors Associated With Acute Ischemic Stroke Door to Needle Time

**DOI:** 10.3389/fneur.2019.00676

**Published:** 2019-06-26

**Authors:** Ferghal McVerry, Annemarie Hunter, Kevin Dynan, Maureen Matthews, Michael McCormick, Ivan Wiggam, Djamil Vahidassr, Fintan McErlean, Mike Stevenson, Emer Hopkins, Jacqueline McKee, James Kelly, Fiona Kennedy, Mark O. McCarron

**Affiliations:** ^1^Stroke Unit and Neurology Department, Altnagelvin Hospital, Derry, United Kingdom; ^2^Neurology Department, Royal Victoria Hospital, Belfast, United Kingdom; ^3^Stroke Unit, Ulster Hospital, Dundonald, United Kingdom; ^4^Stroke Unit, Craigavon Area Hospital, Portadown, United Kingdom; ^5^Stroke Unit, Antrim Area Hospital, Antrim, United Kingdom; ^6^Audit Department, Royal Victoria Hospital, Belfast, United Kingdom; ^7^Centre for Public Health, Queen's University, Belfast, United Kingdom; ^8^Health and Social Care Board, Belfast, United Kingdom; ^9^Stroke Unit, South West Acute Hospital, Enniskillen, United Kingdom

**Keywords:** acute ischemic stroke, thrombolysis, health services research, patient safety, critical care

## Abstract

**Objective:** Pre-hospital, in-hospital, and patient factors are associated with variation in door to needle (DTN) time in acute ischemic stroke (AIS). Publications are usually from large single centers or multicenter registries with less reporting on national results.

**Materials and methods:** All AIS patients treated with intravenous tissue plasminogen activator (iv-tPA) over 4 years (2013–2016) in Northern Ireland were recorded prospectively, including patient demographics, pre-hospital care, thrombolysis rate, and DTN time. Logistic regression was performed to identify factors associated with DTN time.

**Results:** One thousand two hundred and one patients from 10,556 stroke admissions (11.4%) were treated with iv-tPA. Median NIHSS was 10 (IQR 6-17). Median DTN time was 54 min (IQR 36-77) with 61% treated < 60 min from arrival at hospital. National thrombolysis numbers increased over time with improving DTN time (*P* = 0.002). Arrival method at hospital (ambulance OR 2.3 CI1.4-3.8) pre-alert from ambulance (pre-alert OR = 5.3 CI3.5-8.1) and time of day (out of hours, *n* = 650, OR 0.20 CI 0.22-0.38) all *P* < 0.001, were the independent factors in determining DTN time. Variation in DTN time between centers occurred but was unrelated to volume of stroke admissions.

**Conclusion:** Ambulance transport with pre-hospital notification and time of day are associated with shorter DTN time on a national level. Most thrombolysis was delivered outside of normal working hours but these patients are more likely to experience treatment delays. Re-organization of stroke services at a whole system level with emphasis on pre-hospital care and design of stroke teams are required to improve quality and equitable care in AIS nationally.

## Introduction

Reperfusion with intravenous tissue plasminogen activator (iv-tPA) and/or endovascular thrombectomy improves probability of better outcome following acute ischemic stroke (AIS) (1, 2). Patient and systemic factors mean that only a proportion of AIS patients receive thrombolytic treatment (3, 4). The impact of reperfusion is time dependent (5). Guidelines encourage iv-tPA treatment within a 60 min door-to-needle (DTN) time after hospital arrival (6, 7).

Worldwide DTN time is not uniform. Some centers report dramatic reductions in DTN time after service re-organization (8). Elsewhere, improvement in DTN times have been more variable despite dedicated improvement programs (9).

Registries have reported clinical factors associated with DTN time including age, stroke severity, and previous medical history, while hospital factors include hospital size, annual number of thrombolysis cases, and academic status (7, 10). While stroke registries have been associated with improvement in quality of stroke care (11, 12), voluntary stroke registries can be associated with selection bias or may not necessarily reflect nationwide performance. National data on DTN times including both high and low volume centers are limited (13). A key performance index of acute stroke services mandates country-wide assessment of healthcare provision.

The components of the clinical team providing stroke care vary within the week. Dedicated stroke nurses and senior medical staff are routine in normal working hours within UK hospitals but smaller teams with less experience can be tasked with providing iv-tPA outside “office” hours. The impact of varying clinical team make-up and different hospital sites on stroke treatment is unclear.

We performed a country-wide analysis of AIS patients over 4 years in Northern Ireland to measure delays, benchmark DTN time against international recommendations and explore factors influencing DTN times.

## Materials and Methods

### Patients

The study was conducted in Northern Ireland, population 1.86 million with a geographical area of 14,130 km^2^ served by seven lysing stroke centers. All patients who were treated with iv-tPA over four consecutive years from January 1, 2013 to December 31, 2016 were identified from prospective audit logs at each hospital providing acute stroke services. Medical notes and imaging databases were examined retrospectively. Data recorded included age, sex, date of onset of AIS, time of AIS symptom onset (or if not available, time when patient was last known to be well), time/method of arrival at hospital, use of pre-hospital notification, clinical stroke risk factors, and co-morbidities, stroke severity measured using the National Institute for Health Stroke Scale (NIHSS), imaging time and modality, time of administration of iv-tPA, and recording of documented reasons for delay (e.g., clinical fluctuation/diagnostic uncertainty/blood pressure > 185/110 mmHg). Where AIS occurred in someone who was already a hospital inpatient, arrival time was considered to be the same as symptom onset time. An out-of-hours admission was defined as any hospital admission which occurred outside of 09:00–17:00 on Mondays to Fridays, including public holidays. ICD10 codes for number of stroke admissions (ICD10 codes I63-I64) per hospital per year of study were obtained from the department of health. Ethical permission was provided from each stroke center for the study, which was classified as a regional quality improvement audit. As such the Trusts in Northern Ireland waived the need for written informed consent.

### Outcome Measures

Time based measurements were calculated from individual time recordings for each patient as follows:

- Door to needle time = (Time of iv-tPA bolus) – (Time of arrival at hospital)
- Onset to hospital arrival time = (Time of arrival at hospital) – (Time of symptom onset/time last known to be well if onset not witnessed)
- Onset to treatment time = (Time of iv-tPA bolus) – (Time of symptom onset/time last known to be well if onset not witnessed)
- Proportion of AIS patients receiving iv-tPA = [(number receiving iv-tPA/number admissions with AIS at each center per year)] ^*^ 100) (ICD10 code I63-I64).

### Statistical Analysis

Data analysis was performed using SPSS (IBM, Version 19). Statistical analysis included the Mann-Whitney test for binary group comparisons, Kruskal-Wallis ANOVA or Chi-squared test for multiple comparators for ordinal and categorical data where appropriate. A binary logistic regression for factors associated with DTN time of <60 min was performed.

## Results

One thousand, two hundred and one patients received iv-tPA in the study period. There were 10,556 AIS admissions resulting in an overall thrombolysis rate of 11.4% for the 4 year period (Table 1). The mean age of AIS patients treated with iv-tPA was 72 (SD 14) years. Six hundred and fifteen patients (51%) were male. Median NIHSS was 10 (IQR 6-16) and varied significantly between hospitals from 8 to 12 (*P* = 0.0019) (Supplementary Table 1). Median NIHSS measurement reduced from 10 and 11 in 2013 and 2014 to 9 and 8 in 2015 and 2016, respectively (*P* = 0.002) (Table 2).

**Table 1:** Number of thrombolysis treatments, number of admissions with acute ischemic stroke, and thrombolysis rate for each center during audit period.

**Center**	**Thrombolysis cases (*n*)**	**Admissions (*n*)**	**% of admissions treated with thrombolysis**
1	369	2,467	15.0
2	103	947	10.9
3	232	2,494	9.3
4	128	1,378	9.3
5	88	658	13.4
6	179	1,889	9.5
7	102	723	14.1
Total	1,201	10,556	11.4

**Table 2:** Median annualized processing delays and stroke severity.

**Variable (minutes)**	**Year**					
	**All**	**2013**	**2014**	**2015**	**2016**	***P*-value**
*N* (%)	1,201 (100)	277 (23.1)	291 (24.2)	293 (24.4)	339 (28.3)	-
Door to needle time (IQR)	54 (36–77)	64 (41–88)	52 (35–76)	53 (38–72)	51 (34–72)	<0.001
NIHSS (IQR)	10 (6–16)	10 (6–16)	11 (7–18)	9 (6–16)	8 (5–15)	0.002
Onset to treatment (IQR)	140 (105–182)	145 (110–187)	126 (98–171)	145 (108–185)	135 (101–180)	0.019
Onset to arrival (IQR)	78 (53–111)	73 (48–102)	72 (49–104)	81 (61–119)	83 (55–116)	0.001
DTN time^*^ <60 min	707/1,176 (60.1%)	130/274 (47.4%)	179/289 (61.9%)	187/289 (64.7%)	211/324 (65.1%)	0.001

*IQR, interquartile range; NIHSS, National Institute of Health Stroke Score; DTN, Door-to-Needle*.

* *DTN Time not available for 24 patients and year of treatment not available for 1*.

**Figure 1:**
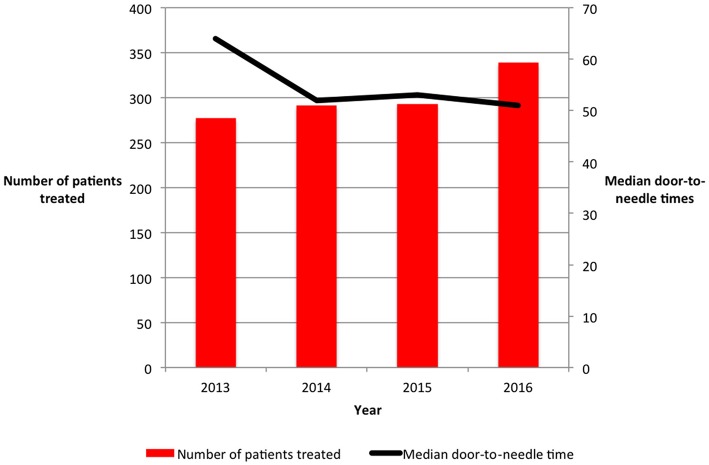
Annual number of patients with acute ischemic stroke treated with intravenous thrombolysis and median door-to-needle times in Northern Ireland from 2013 until 2016.

The number of AIS patients treated with iv-tPA per year increased, from 277 patients in 2013 to 339 patients in 2016 (Figure 1). This resulted in the thrombolysis rate increasing from 10.7% in 2013 to 12.4% in 2016 (*P* = 0.028). The number of patients treated with iv-tPA varied between centers, ranging from 88 cases to 369 cases. The thrombolysis rate within each center ranged from 9.3 to 15.0% (Table 1).

Median DTN time was 54 min (IQR 36-77). A significant reduction in DTN time occurred over the study period, reducing from 64 min in 2013 to 51 min in 2016 (*P* < 0.001). 60.1% of AIS patients were treated with a DTN time of < 60 min. The percentage of patients treated within 60 min of arrival in hospital increased from 47.4% in 2013 to 65.1% in 2016 (*P* < 0.001). Median onset to treatment time (OTT) time reduced from 145 min in 2013 to 135 min in 2016 (*P* = 0.019), although median OTT times in 2013 and 2015 were similar. Onset to hospital time was significantly longer in 2015 and 2016 than 2013 and 2014 (*P* = 0.001, Table 2).

Median DTN time varied between centers. The center with the shortest DTN times was center 7, and center 3 had the longest DTN times (*P* < 0.001) (Table 1). Arrival at hospital via ambulance with pre-hospital notification was associated with shorter in-hospital DTN time than use of ambulance without pre-hospital notification. Other arrival methods or in-patients experiencing AIS were associated with longer DTN times (*P* < 0.001) (Table 3). Onset to arrival time and OTT varied significantly between centers (*P* < 0.001 and *P* = 0.006, respectively, Table 1).

**Table 3:** Door-to-needle time according to arrival method.

**Method of arrival**	**N**	**Door to needle time in minutes median (IQR)**
Other/inpatient/transfer	138	77 (55–113)
Ambulance no pre-alert	181	65 (46–93)
Ambulance with pre-alert	858	49 (34–68)

*Door to needle time not recorded for 24 cases*.

**Figure 2:**
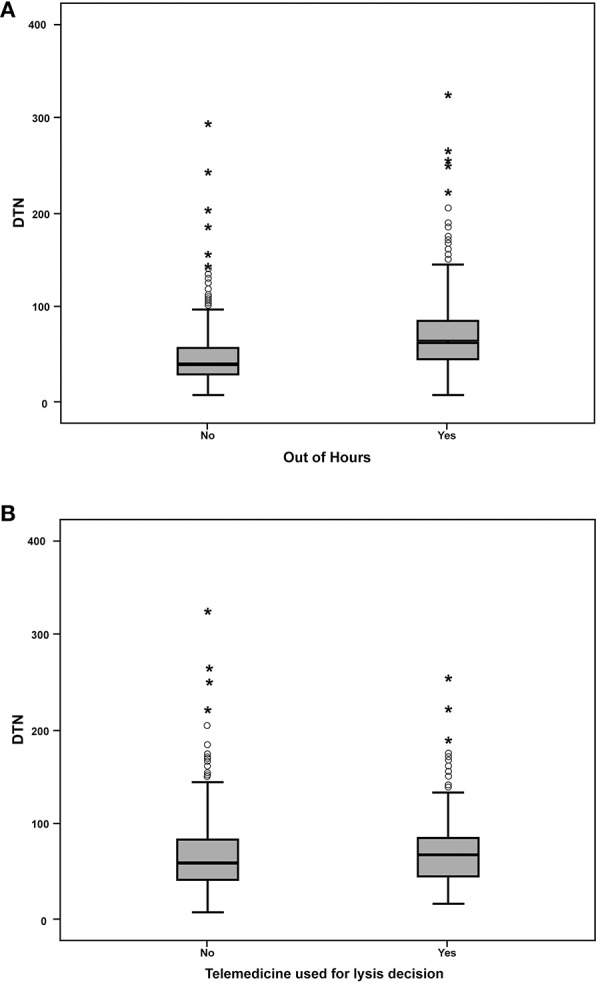
Box-whisker plots. **(A)** Door-to-needle times for acute ischemic stroke patients treated with intravenous thrombolysis in hours (09:00–17:00) compared with patients treated out of hours. **(B)** Comparison of out of hours door-to-needle times with and without telemedicine.

Fifty-eight percent of patients (*n* = 698) received iv-tPA outside of typical (09:00–17:00) office hours. Patients treated out-of-hours had significantly longer median DTN times than those patients treated between 09:00 and 17:00 (63 and 41 min, respectively, *P* < 0.001, Figure 2A).

For patients treated out-of-hours, use of telemedicine consultation was associated with longer median DTN times than face-to-face consultation (*n* = 142, median 68 min, IQR 54-86 and *n* = 556, median 60 min, IQR 43-84, respectively (*P* = 0.002, Figure 2B).

Factors associated with the likelihood for achieving DTN <60 min were identified from binary logistic regression and included: in-hours vs. out-of-hours (OR = 0.232 CI 0.173-0.307 *P* < 0.001), arrival with pre-hospital alert or arrival by ambulance (OR = 5.342 CI 3.472-8.219, *P* < 0.001 and OR = 2.168 CI 1.288-3.650, *P* = 0.004, respectively), year of treatment (OR = 2.318 CI = 1.598-3.364, *P* < 0.001), and admitting hospital (OR = 3.518, CI 1.931-6.410, *P* < 0.001). The most significant factor was treatment occurring in-hours rather than out-of-hours, followed by arrival method. Year of treatment and site of treatment had smaller impacts on DTN time than both arriving in-hours and arriving with pre-hospital notification (Table 4).

**Table 4:** Binary logistic regression analysis for door-to-needle processing times under 60 min (results relative to 2013, center results relative to center 1).

**Variable**	**Odds Ratio**	**Confidence Interval**	***P-*value**
Out of hours admission	0.23	0.17–0.31	<0.001
Use of ambulance	2.17	1.29–3.65	0.004
Use of ambulance with pre-alert	5.34	3.45–8.22	<0.001
2014	2.02	1.39–2.94	<0.001
2015	2.13	1.46–3.11	<0.001
2016	2.32	1.60–3.36	<0.001
Center 3	0.58	0.36–0.84	0.004
Center 7	3.52	1.93–6.41	<0.001

**Figure 3:**
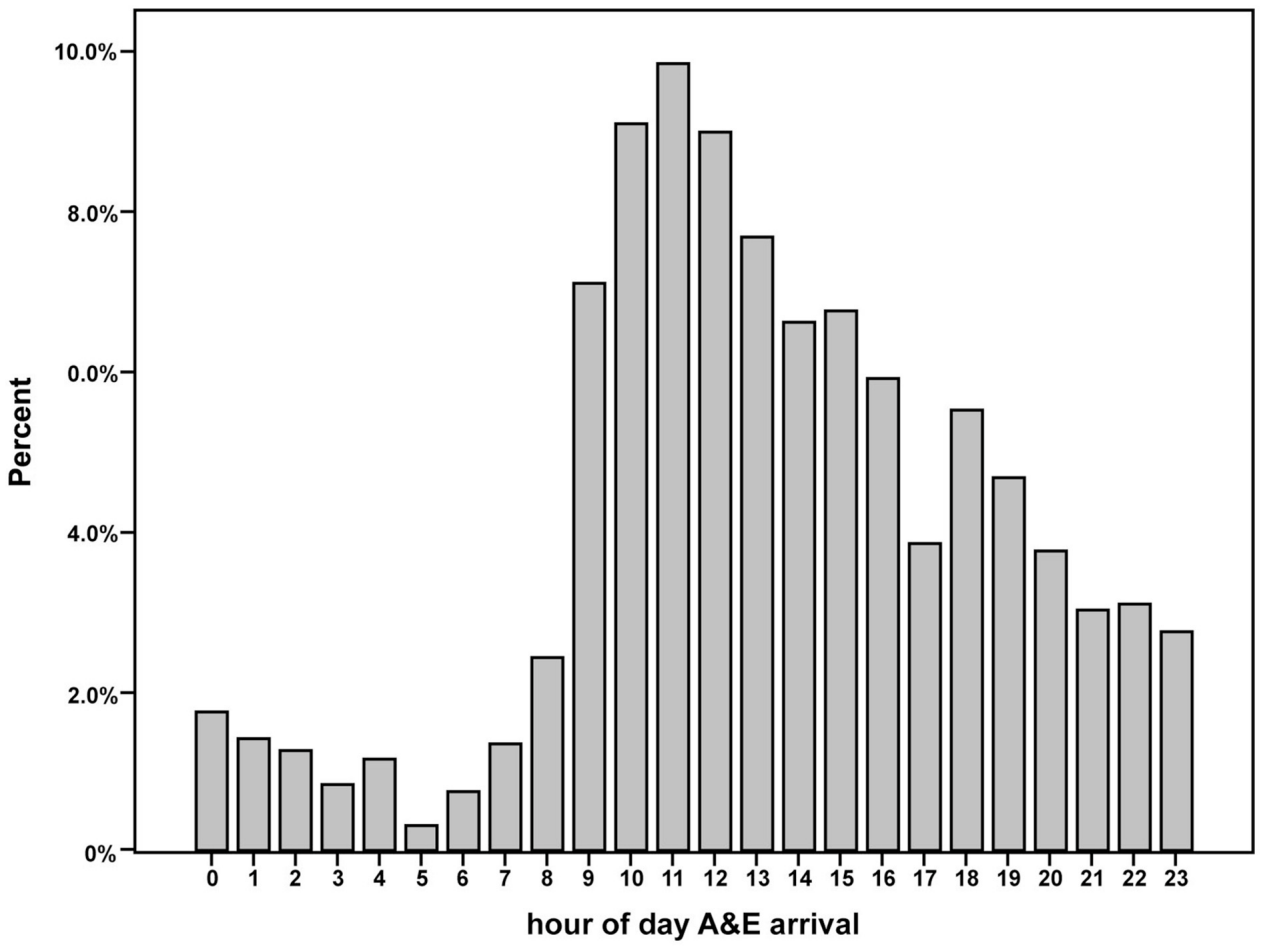
Hourly admission numbers of patients with acute ischemic stroke treated with intravenous thrombolysis therapy.

When assessed according to a 24 h clock period, patients arrived at hospital with AIS symptoms at each hourly period, but the distribution of patient arrival times varied substantially with a peak around late morning and early afternoon. The smallest proportion of patients arrived after midnight and declined until 06:00–07:00 and increased thereafter (Figure 3).

## Discussion

This national study demonstrated incremental improvements in DTN times and numbers of patients treated with iv-tPA. Ambulance transport with pre-alert for hospital stroke teams during office working hours shortened hospital delays to treatment with thrombolysis. The absolute number of patients receiving iv-tPA for AIS nationally per year increased by 22%, from 277 in 2013 to 339 in 2016. The highest proportion of patients receiving thrombolysis treatment was seen in the hospital with the largest absolute number of stroke admissions and iv-tPA administrations. This finding is consistent with international trends (7). However, smaller hospitals with fewer overall AIS patient admissions and thrombolysis cases were able to achieve high thrombolysis rates and fast DTN times suggesting that variation is not solely related to size of hospital or catchment area. Motivation and benchmarking results may facilitate fast DTN times. Furthermore, the center with the shortest median DTN times in this study was one of the smallest centers in terms overall stroke patient admissions and number of AIS patients treated with iv-tPA.

Factors associated with DTN time were not restricted to hospital size or location. The single factor with most impact on odds for achieving DTN times of ≤60 min was the time of day that each patient required treatment. Patients with AIS treated out of normal working hours were less likely to receive timely access to iv-tPA. Importantly, this group constituted the majority of patients (58%) who were treated with iv-tPA.

The members of a stroke thrombolysis team within office hours can be very different to other parts of the week. For example, a dedicated stroke nurse with experience in delivering iv-tPA as part of a multidisciplinary team is present in most hospitals during usual “office hours” while nursing staff with less thrombolysis experience and other competing clinical commitments are more likely to attend at other times. Senior medical staff with expertise in stroke management may not be present for referrals of AIS patients received out-of-hours but are often present during office hours. Emergency department and radiology department staffing levels also show variation according to time of day and each of these departments plays a critical role in the AIS thrombolysis pathway.

Given the critical importance of time to AIS thrombolysis treatment in influencing clinical outcome, the variation in staff levels, and experience according to time of day has important implications. The Helsinki model provided a dedicated and constant staffing resource and avoided out-of hours variation in DTN times (14). They introduced 12 measures to reduce DTN times. As approximately 80% of AIS patients in our study were admitted between 08:00 and 22:00, provision of dedicated stroke teams for longer periods of the day and at weekends requires consideration. Time of day has been associated with DTN time variability in some other populations but not all (15–18). Single centers have reported improvement in DTN time in hours and out of hours using the Helsinki model but translating that improvement to a national level across a number of sites has not been reported (19). Other international models of care have shown variation in lysis rates and treatment times (13).

Telemedicine is used in hospitals to permit remote clinical assessment of AIS by a senior clinician and has a proven evidence base for increasing access to iv-tPA treatment (20). Four of our centers used telemedicine for some of their out-of-hours thrombolysis service, permitting further analysis. This study has shown that telemedicine use can be associated with longer DTN times compared with other methods during out of hours, suggesting that local pathways involving telemedicine which employ sequential assessments rather than simultaneous assessments (e.g., remote assessment after CT Brain scan rather than during the Emergency room and/or CT period) require alterations to minimize delays. Further experience at local hospital level with dedicated stroke leaders offers opportunities to quality-improve this aspect of iv-tPA delivery for AIS patients.

Pre-hospital factors had an important influence on subsequent in-hospital performance and overall time to treatment. DTN times improved over the 4 year period, but there was a significant trend toward longer OTH time in 2016 compared to 2013. This had a resultant effect on prolonging OTT time, which is the most relevant measure from the individual patient perspective. Logically any delay in pre-hospital time will counteract the benefits achieved through reducing in-hospital DTN time.

Lack of improvement in pre-hospital triage times have been recorded elsewhere and pose a challenge for stroke service delivery (21). Novel solutions to pre-hospital care such as mobile stroke ambulances equipped to administer iv-tPA for AIS patients are not yet widespread and so far have been limited to large urban populations (12, 21). A stroke emergency mobile unit has shown faster and more lysis treatments than conventional care (12). Patients, who were recognized by ambulance teams as potentially presenting as a stroke and had provided advanced notification to hospital (“pre-alert”), benefitted from shorter DTN times compared to patients without pre-alert or other presentations. The impact of pre-hospital services is crucial both in reducing OTT time and influencing in-hospital processes.

Stroke severity scores were consistent with the known trial and meta-analysis data supporting iv-tPA use in AIS (5). There was a trend toward lower NIHSS measurement in treated cases over time. Longer OTH times with increasing numbers of treated AIS patients may reflect the fact that patients with less severe AIS take longer to arrive in hospital (22). The increasing numbers of iv-tPA-treated patients may suggest increasing confidence among clinicians in recognizing and treating AIS. In addition, the department of health and individual health trusts or hospitals have encouraged delivery of thrombolysis for AIS patients.

Several clinical factors such as past medical history, co-morbidities, hypertension, and treatment with medication such as anticoagulants can result in treatment delay for individual patients (23). These factors were all recorded for this study but did not have a significant impact on treatment times on a national level. Therefore, while individual factors can and will continue to influence treatment time for individual patients, such factors have minimal impact at a population level.

Limitations of this study include lack of recorded outcome after thrombolysis treatment but as our focus was on assessing factors associated with delayed treatment this was not a requirement. This was a retrospective study and for a small number of patients a specific DTN time measurement was not recorded due to missing data, but this was a small proportion of all cases (2%). This study has several strengths. We have reported on all AIS patients treated with iv-tPA on a national level. Previous registry data publications from selected centers may be associated with selection bias. Other registries suffer from “inflation bias” where cases associated with known reasons for delay are specifically not recorded and this can influence how reported DTN times appear (23, 24). Some other centers have published improvement in-hours only, which may not reflect care given to the majority of stroke admissions (8). The large number of patients in this study permitted the use of a binary logistic regression analysis permitting a ranking for factors most strongly associated with DTN time, namely out of hours and pre-hospital stroke care, and permit consideration of alternative models for stroke thrombolysis administration on a national level. Endovascular thrombectomy is now an established form of treatment for AIS with larger artery occlusion. Northern Ireland has an endovascular center, which treats such patients with or without iv-tPA and relies on early alert of eligible patients (25). Stroke physicians now frequently lyse AIS patients at a stroke center and transfer those patients with major vascular occlusions to the endovascular center while iv-tPA infusions are running.

This study has demonstrated national trends in AIS lysis treatment and DTN time over a 4 year period. An increasing absolute number and proportion of AIS patients were treated with iv-tPA over time and this occurred with an incremental decline in DTN time. Variation in treatment times was associated with time of day and arrival method, year, and admitting hospital but large volumes of AIS admissions were not a pre-requisite for short DTN time. Specific focus on out of hours and pre-hospital services are required to improve national DTN times and reduce inequality of access to thrombolysis for all AIS patients (26). This type of study will facilitate quality-improvement interventions to continue to improve the number of lysed AIS patients and their treatment times on a national level.

## Data Availability

The datasets generated for this study are available on request to the corresponding author.

## Ethics Statement

The study qualified as an audit. Each Trust approved the study and declared formal ethics approval was not required.

## Author Contributions

FeM designed the study, collected data, and wrote the first draft. AH, KD, MauM, MicM, IW, DV, JM, JK, and FK collected data and revised drafts of the paper. FiM helped administer the study and revised drafts of the paper. MS performed the statistical analyses and revised drafts of the paper. EH collected data for the paper and revised drafts. MOM designed the study, coordinated data collection, and revised drafts of the paper.

### Conflict of Interest Statement

The authors declare that the research was conducted in the absence of any commercial or financial relationships that could be construed as a potential conflict of interest.

## References

[1] Wardlaw JM Murray V Berge E del Zoppo GJ Thrombolysis for acute ischaemic stroke. Cochrane Database Syst Rev. (2014) CD000213. 10.1002/14651858.CD000213.pub3 PMC4153726 25072528

[2] Badhiwala JH Nassiri F Alhazzani W Selim MH Farrokhyar F Spears J. Endovascular thrombectomy for acute ischemic stroke. JAMA. (2015) 314:1832. 10.1001/jama.2015.13767 26529161

[3] Paul CL Ryan A Rose S Attia JR Kerr E Koller C. How can we improve stroke thrombolysis rates? A review of health system factors and approaches associated with thrombolysis administration rates in acute stroke care. Implement Sci. (2016) 11:51. 10.1186/s13012-016-0414-6 27059183 PMC4825073

[4] Tawil S El Cheripelli B Huang X Moreton F Kalladka D MacDougal NJ. How many stroke patients might be eligible for mechanical thrombectomy? Eur Stroke J. (2016) 1:264–71. 10.1177/2396987316667176 31008287 PMC6301245

[5] Emberson J Lees KR Lyden P Blackwell L Albers G Bluhmki E. Effect of treatment delay, age, and stroke severity on the effects of intravenous thrombolysis with alteplase for acute ischaemic stroke: a meta-analysis of individual patient data from randomised trials. Lancet. (2014) 384:1929–35. 10.1016/S0140-6736(14)60584-5 25106063 PMC4441266

[6] Saver JL. Time is brain–quantified. Stroke. (2006) 37:263–6. 10.1161/01.STR.0000196957.55928.ab 16339467

[7] Saver JL Smith EE Fonarow GC Reeves MJ Zhao X Olson DM. The golden hour and acute brain ischemia: presenting features and lytic therapy in >30,000 patients arriving within 60 minutes of stroke onset. Stroke. (2010) 41:1431–9. 10.1161/STROKEAHA.110.583815 20522809 PMC2909671

[8] Meretoja A Strbian D Mustanoja S Tatlisumak T Lindsberg PJ Kaste M. Reducing in-hospital delay to 20 minutes in stroke thrombolysis. Neurology. (2012) 79:306–13. 10.1212/WNL.0b013e31825d6011 22622858

[9] Fonarow GC Zhao X Smith EE Saver JL Reeves MJ Bhatt DL Door-to-needle times for tissue plasminogen activator administration and clinical outcomes in acute ischemic stroke before and after a quality improvement initiative. JAMA. (2014) 90095:1632–40. 10.1001/jama.2014.3203 24756513

[10] Fonarow GC Smith EE Saver JL Reeves MJ Bhatt DL Grau-Sepulveda M V. Timeliness of tissue-type plasminogen activator therapy in acute ischemic stroke patient characteristics, hospital factors, and outcomes associated with door-to-needle times within 60 minutes. Circulation. (2011) 123:750–8. 10.1161/CIRCULATIONAHA.110.974675 21311083

[11] Ormseth CH Sheth KN Saver JL Fonarow GC Schwamm LH. The American Heart Association's get with the guidelines (GWTG)-stroke development and impact on stroke care. Stroke Vasc Neurol. (2017) 2:94–105. 10.1136/svn-2017-000092 28959497 PMC5600018

[12] Ebinger M Winter B Wendt M Weber JE Waldschmidt C Rozanski M. Effect of the use of ambulance-based thrombolysis on time to thrombolysis in acute ischemic stroke. JAMA. (2014) 311:1622–31. 10.1001/jama.2014.2850 24756512

[13] Liu Q Ranta AA Abernethy G Barber PA. Trends in New Zealand stroke thrombolysis treatment rates. N Z Med J. (2017) 130:50–6. 28384147

[14] Curtze S Meretoja A Mustanoja S Putaala J Lindberg T Leppa M. Does time of day or physician experience affect outcome of acute ischemic stroke patients treated with thrombolysis? A study from Finland. Int J Stroke. (2012) 7:511–6. 10.1111/j.1747-4949.2012.00795.x 22494345

[15] Fang K Churilov L Weir L Dong Q Davis S Yan B. Thrombolysis for acute ischemic stroke: do patients treated out of hours have a worse outcome? J Stroke Cerebrovasc Dis. (2014) 23:427–32. 10.1016/j.jstrokecerebrovasdis.2013.03.029 23635920

[16] Bray BD Cloud GC James MA Hemingway H Paley L Stewart K. Weekly variation in health-care quality by day and time of admission: a nationwide, registry-based, prospective cohort study of acute stroke care. Lancet. (2016) 388:170–7. 10.1016/S0140-6736(16)30443-3 27178477

[17] Khor M Bown A Barrett A Counsell C MacLeod M-J Reid J. Pre-hospital notification is associated with improved stroke thrombolysis timing. J R Coll Physicians Edinb. (2015) 45:190–5. 10.4997/JRCPE.2015.303 26517096

[18] Lorenzano S Ahmed N Tatlisumak T Gomis M Davalos A Mikulik R. Within-day and weekly variations of thrombolysis in acute ischemic stroke: results from safe implementation of treatments in stroke-international stroke thrombolysis register. Stroke. (2014) 45:176–84. 10.1161/STROKEAHA.113.002133 24262329

[19] Wu TY Coleman E Wright SL Mason DF Reimers J Duncan R. Helsinki stroke model is transferrable with “real-world” resources and reduced stroke thrombolysis delay to 34 min in Christchurch. Front Neurol. (2018) 9:290. 10.3389/fneur.2018.00290 29760676 PMC5937050

[20] Johansson T Wild C. Telemedicine in acute stroke management: Systematic review. Int J Technol Assess Health Care. (2010) 26:149–55. 10.1017/S0266462310000139 20392317

[21] Pulvers JN Watson JDG. If time is brain where is the improvement in prehospital time after stroke? Front Neurol. (2017) 8:617. 10.3389/fneur.2017.00617 29209269 PMC5701972

[22] McCarron MO Armstrong M McCarron P. Potential for quality improvement of acute stroke management in a district general hospital. Emerg Med J. (2008) 25:270–3. 10.1136/emj.2007.051680 18434459

[23] Kamal N Sheng S Xian Y Matsouaka R Hill MD Bhatt DL. Delays in door-to-needle times and their impact on treatment time and outcomes in get with the guidelines-stroke. Stroke. (2017) 48:946–54. 10.1161/STROKEAHA.116.015712 28228574

[24] Smith EE Saver JL Alexander DN Furie KL Hopkins LN Katzan IL. Clinical performance measures for adults hospitalized with acute ischemic stroke. Stroke. (2014) 45:3472–98. 10.1161/STR.0000000000000045 25256184

[25] Nixon AM Jamison M Rennie IM Flynn PA Smyth G Wiggam I. ESCAPE to reality, post-trial outcomes in an ESCAPE centre: a retrospective case-control study. Ulster Med J. (2018) 87:22–6. 29588552 PMC5849948

[26] Kobayashi A Czlonkowska A Ford GA Fonseca AC Luijckx GJ Korv J. European Academy of Neurology and European Stroke Organization consensus statement and practical guidance for pre-hospital management of stroke. Eur J Neurol. (2018) 25:425–33. 10.1111/ene.13539 29218822

