# Supplementary material for: Country-Wide Analysis of Systemic Factors Associated With Acute Ischemic Stroke Door to Needle Time
Source: Front Neurol. 2019 Jun 26;10:676. doi: 10.3389/fneur.2019.00676 (PMC6606974; doi:10.3389/fneur.2019.00676)
Supplement: Supplementary file 1 [file Table_1.docx]

|  | Hospital Site | | | | | | | *P* value |
| --- | --- | --- | --- | --- | --- | --- | --- | --- |
| Variable | 1 | 2 | 3 | 4 | 5 | 6 | 7 |  |
| N  (%) | 369  (30.7) | 103  (8.6) | 232  (19.3) | 128  (10.7) | 88  (7.3) | 179  (14.9) | 102  (8.5) | - |
| DTN  (IQR) | 49  (35-70) | 55  (33-83 | 61  (41-88) | 55  (41-76) | 58  (34-81) | 59  (42-86) | 33  (19-56) | <0.001 |
| NIHSS  (IQR) | 10  (6-16) | 11  (7-18) | 10  (6-16) | 10  (6-17) | 12  (6-18) | 8  (5-15) | 9.5  (6-17) | 0.019 |
| Onset to Bolus  (IQR) | 135  (98-180) | 126 (100-162) | 156 (120-197 | 127 (105-169) | 139 (101-175) | 145 (115-197) | 123  (90-155) | <0.001 |
| Onset to Arrival (IQR) | 75  (50-115) | 73  (47-95) | 88  (63-123) | 72  (55-93) | 75.5  (56-104) | 78  (55-112) | 82  (50-118) | 0.006 |
| DTN < 60mins  (%)* | 233/353  (66.0%) | 56/101  (55.4%) | 113/227  (49.8%) | 93/128  (64.8%) | 46/88  (52.3%) | 93/178 (52.2%) | 83/102  (81.4%) | <0.001 |

Supplementary Table 1. Audit results according to hospital site

DTN=door-to Needle, IQR= interquartile range. (*DTN time was not available for 24 patients).
